# An Ultrastructural and Immunohistochemical Analysis of the Outer Plexiform Layer of the Retina of the European Silver Eel (*Anguilla anguilla* L)

**DOI:** 10.1371/journal.pone.0152967

**Published:** 2016-03-31

**Authors:** Jan Klooster, Maarten Kamermans

**Affiliations:** 1 Retinal Signal Processing Lab, Netherlands Institute for Neuroscience, Amsterdam, The Netherlands; 2 Department of Neurogenetics, Academic Medical Center, University of Amsterdam, Amsterdam, The Netherlands; Universidade Federal do ABC, BRAZIL

## Abstract

Here we studied the ultrastructural organization of the outer retina of the European silver eel, a highly valued commercial fish species. The retina of the European eel has an organization very similar to most vertebrates. It contains both rod and cone photoreceptors. Rods are abundantly present and immunoreactive for rhodopsin. Cones are sparsely present and only show immunoreactivity for M-opsin and not for L-, S- or UV-cone opsins. As in all other vertebrate retinas, Müller cells span the width of the retina. OFF-bipolar cells express the ionotropic glutamate receptor GluR4 and ON-bipolar cells, as identified by their PKCα immunoreactivity, express the metabotropic receptor mGluR6. Both the ON- and the OFF-bipolar cell dendrites innervate the cone pedicle and rod spherule. Horizontal cells are surrounded by punctate Cx53.8 immunoreactivity indicating that the horizontal cells are strongly electrically coupled by gap-junctions. Connexin-hemichannels were found at the tips of the horizontal cell dendrites invaginating the photoreceptor synapse. Such hemichannels are implicated in the feedback pathway from horizontal cells to cones. Finally, horizontal cells are surrounded by tyrosine hydroxylase immunoreactivity, illustrating a strong dopaminergic input from interplexiform cells.

## Introduction

The genus *Anguila* consists of 18 species that are widely distributed throughout the world. The American eel (*Anguilla rostrata*), the Japanese eel (*Anguilla japonica*) and the European eel (*Anguilla anguilla*) migrate over long distance [1]. This study focuses on the European eel. European eels spawn in the Sargasso Sea. After hatching the larvae migrate across the Atlantic Ocean to the European coastal waters over about a 6 month period [1]. The European eel undergoes metamorphosis twice in its life. First, the larvae metamorphose into glass eels and move inwards to freshwater rivers and lakes. There they stay for several years to become yellow eels [1]. In the second metamorphosis, the yellow eels change to silver eels in preparation for the transatlantic journey back to the Sargasso sea where they spawn and die [2,3].

During their life cycle, the European eels migrate from deep ocean waters to shallow fresh water lakes and back again. Such large changes in environment requires major adaptation of their sensory organs [4]. One of the sensory organs where such a change is clearly visible is the retina. Since the spectral composition of the light in deep ocean waters and shallow fresh water lakes differs considerably, one expects changes in the properties of the photoreceptors during metamorphosis.

Photoreceptors are the light sensitive cells in the retina. Opsins present in the outer segments (OS) of the photoreceptors, mediate the conversion of photons into an electrochemical signal. There are two types of photoreceptors; rods for low light level vision and cones high light level vison. Both photoreceptor types are present in the retina of the European eel [2]. Cones might come in different types depending on the opsin they express. Apart from expressing different opsins, the spectral sensitivity of the opsins can be somewhat tuned by the type of retinaldehyde chromophore it is covalently bound to. For opsins bound to the A2 chromophore the λ_max_ occurs at a longer wavelength than if it was bound to the A1 chromophore [5,6]. This shift happens during the silvering process of the European eel [7,8].

The signals of the photoreceptors are further processed by the retinal network. The retina is a layered structure with two synaptic layers and three nuclear layers. Photoreceptor somata are located in the first nuclear layer is the outer nuclear layer (ONL). At the first synaptic layer, the outer plexiform layer (OPL) the photoreceptors make synaptic contacts with horizontal cells and bipolar cells. The somata of horizontal, bipolar and amacrine cells are located in the inner nuclear layer (INL). In the second synaptic layer, the inner plexiform layer (IPL), bipolar, amacrine and ganglion cells make synaptic contacts. The somata of the ganglion cells are located in the ganglion cell layer (GCL). The ganglion cells transfer the signal to the optic tectum.

Currently there are no immunohistochemical studies examining the European silver eel retinal organization at either the light- or electronmicroscopical level. Here we use these techniques to address three aspects of the organization of the outer retina of the European silver eel.

1. In many fish species different spectral types of cones are present in the retina. In the retina of the European yellow eel two types of cones with peak sensitivities at 540–545 nm (green sensitive) and 435-440nm (blue sensitive) respectively have been described [7,9,10]. The blue sensitive cones are sparsely observed [9], however it is currently unknown if this is also the case in the European sliver eel [7].
2. In the outer retina, the visual information streams into two parallel pathways, the ON- and OFF-pathways [11]. In the dark photoreceptors release L-glutamate that depolarizes horizontal cells and OFF-bipolar cells by activation of an ionotropic glutamate receptor [12] and hyperpolarizes ON-bipolar cells by activation of a metabotropic glutamate receptor [13]. The subunit composition of the ionotropic glutamate receptors on bipolar cells seems to vary between different species [14]. It is currently unknown if the retina of the European silver eel is also organized in this manner.
3. The center/surround organization of the receptive fields first occurs in the outer retina due to the activity of horizontal cells [15]. Horizontal cells are strongly electrically coupled by gap-junctions [16] and feedback to photoreceptors [17]. Connexins form both the gap-junctions involved in the electrical coupling of the horizontal cells and the hemichannels important for the negative feedback pathway from horizontal cells to photoreceptors [18]. As for the glutamate receptors, the specific type of connexins present varies between species. If the retina of the European silver eel is organized in such a way is presently unknown.

In the present study we show that the European silver eel, expresses rhodopsin in rods and M-opsin in cones. Horizontal cells are coupled by gap junctions and express hemichannels at their dendrites. The gap-junctions and hemichannels are composed of a connexin homologue to the carp Cx53.8. OFF-bipolar cells express GluR4 and ON-bipolar cells express mGluR6 and PKCα at their dendrites. This synaptic organization is very similar to that of cyprinid fish except that the European silver eel has only one cone type.

## Material and Methods

### Animals

Hatchery-raised eels that had metamorphosed to the silver eel stage (~30–35 cm long) were kindly provided by a commercial fish company, Klooster BV (Volmolen 8, Enkhuizen, The Netherlands). The eyes used here were collected in September and October from animals euthanized by decapitation for human consumption. As such no approval from an ethical committee was required.

### Lightmicroscopy

Eyes were isolated and fixated. For immunohistochemistry eyes were fixed by immersion in 4% paraformaldehyde (PFA) phosphate buffered at pH 6.5 for 10 min, then in 4% PFA carbonate buffered at pH 10.4 for 10 min. The eye’s anterior segment and lens were removed, the eyes were rinsed in phosphate buffer (PB, pH7.4), and cryoprotected in 12,5% sucrose in PB for 30 min, followed by 25% sucrose in PB for 1 to 2 hours. Eyes were placed in Tissue Tex and frozen in an aluminum boat. 10 μm frozen sections were made and kept at -20°C until used. Retinal sections were washed three times (5 min each) in PB saline (PBS), and blocked in 2% normal goat serum (NGS) in PBS for 20 min. Sections were then incubated for 24 hours with primary antibodies against glutamine synthetase (1:200), M-opsin (1:200) [19], Calretinin (1:200), Tyrosine Hydroxylase (1:200), Cx53.8 (1:1000) [20], GluR4 (1:10), PKCα (1:200), mGluR6 (1:200) [21] followed by three 5 min PBS washing steps. Next, the sections were incubated with goat anti mouse Alexa 488 or goat anti rabbit Alexa 488. In double label experiments sections were incubated in a mixture of primary antibodies and followed by incubation with a mixture of the appropriate secondary antibodies. Sections were coverslipped with Vectashield containing propidium iodide, and observed on inverted Zeiss Axiovert 100M microscope equipped with the LSM 510 Meta laser scanning confocal module. The double label experiment with mGluR6 and PKCα antibodies was examined on a Leica SP 5 microscope. Data are presented as a single optical slice with z thickness of 0.4 μm.

### Standard Electronmicroscopy

For standard electronmicroscopy (EM) eyes were fixed in 4% PFA and 2% glutaraldehyde in PB for 24 hours. The anterior eye segment was removed, the posterior eye segment was rinsed in 0.1 M sodium cacodylate buffered at pH 7.4 and postfixed for 2 hours in 1% OsO_4_ in 0.1 M sodium cacodylate buffer (pH 7.4), containing 1.5% potassium ferricyanide. The posterior eye segments were dehydrated and embedded in epoxy resin. 60 nm sections were cut, counterstained with uranyl acetate and lead citrate and examined in a FEI Tecnai electronmicroscope.

### Immune Electronmicroscopy

For the immunohistochemical analysis at the EM level, the same fixation and cryprotection procedures were used as described for lightmicroscopical analysis. 40 μm frozen sections were made on a freezing microtome and collected in PB. Retinal sections were incubated for 48 hours with antisera against Cx53.8 (1:1000), GluR4 (1:10), PKCα (1:200), mGluR6 (1:200). After rinsing the sections were incubated in a Bright vision poly HRP anti rabbit IgG or Bright vision poly HRP anti mouse IgG. To visualize the peroxidase, the sections were incubated in a Tris-HCl diaminobenzidine (DAB) solution containing 0.03% H_2_O_2_. The DAB reaction product was intensified by the gold substituted silver peroxidase method [22]. Sections were rinsed in sodium cacodylate buffer 0.1 M (pH 7.4) and postfixed for 20 min in 1% OsO_4_ supplemented with 1% potassium ferricyanide in sodium cacodylate buffer 0.1 M (pH 7.4). After rinsing in sodium cacodylate buffer, the material was dehydrated and embedded in epoxy resin. Ultrathin sections were made and examined in a FEI Tecnai 12 microscope. Pictures were taken as tiff files and processed by Adobe Photoshop CS4.

### Antibodies

Details about the antibodies used can be found in Table 1.The antibodies against Calretinin, Tyrosine Hydroxylase, PKCα, GluR4, mGluR6 are widely used in vertebrate retina research. Th e Cx53.8 antibody is a marker for horizontal cell gap junctions in fish retina [20]. The opsin antibodies are raised against the sequences of the zebrafish opsins, when positive in the eel retina, they stained the discs of the photoreceptor, demonstrating their validity. The GluR1 antibody from Chemicon (AB1504), the monoclonal antibody against GluR2 from Chemicon (MAB 397) and NMDA2B (AB1557) antibody from Chemicon did not give a positive result in our hands. Also the Cx55.5 and Cx52.6 antibodies raised in our lab did not work in the retina of the European silver eel.

**Table 1:** Antibodies used.

Protein	Raised in	Raised against	Type	Source
L-opsin	Rabbit	C-terminus zebrafish L-opsin	Polyclonal	David R Hyde lab
M-opsin	Rabbit	C-terminus zebrafish M-opsin	Polyclonal	David R Hyde lab
S-opsin	Rabbit	C-terminus zebrafish S-opsin	Polyclonal	David R Hyde lab
UV-opsin	Rabbit	C-terminus zebrafish UV-opsin	Polyclonal	David R Hyde lab
Rhodopsin	Rabbit	C-terminus zebrafish rhodopsin	Polyclonal	David R Hyde lab
Calretinin	Rabbit	Rat calretinin	Polyclonal	AB 5054 Chemicon Lot NG1780667
Tyrosine Hydroxylase	Mouse	Recognizes an epitope on the outside of the regulatory N-terminus	Monoclonal	MAB318 Chemicon; Lot NG1723872
cpCx53.8	Rabbit	MILELSSIMKK of carp Cx53.8	Polyclonal	Janssen-Bienhold lab
GluR4	Rabbit	C-Terminus of GluR4	Polyclonal	AB1508 Chemicon; lot 2070825
PKCα	Mouse	296–317 amino acid sequence	Monoclonal	MC5 P-5074 Sigma; Lot 093K4855
mGluR6	Rabbit	QKSSDKQGETKVEPDRSQ 899–905 zfmGlur6b	Polyclonal	Neuhauss lab
Glutamine synthetase	Mouse	Human glutamine synthetase aa1-373	Monoclonal	BD Transduction lab; cat 610518

## Results

**Fig 1:**
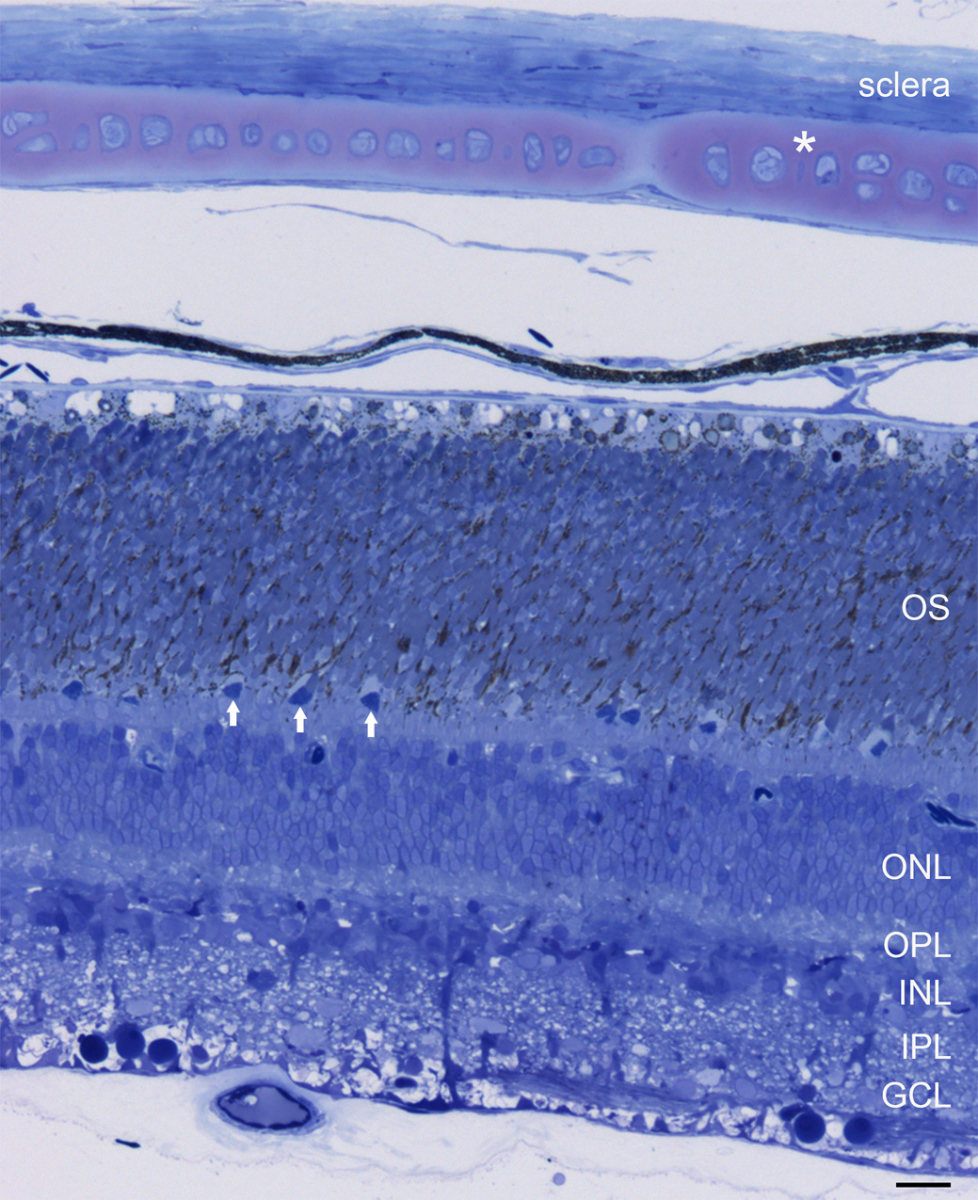
Lightmicrograph of a 1 μm thick section of the posterior eye segment of the European eel. The tissue is embedded in epoxy resin, stained with toluidine blue. From top to bottom one can identify the sclera, the outer segments of the photoreceptors (OS), the inner segments of the cones (arrows), the outer nuclear layer (ONL), the outer plexiform layer (OPL), the inner nuclear layer (ONL), the inner plexiform layer (IPL and the ganglion cell layer (GCL). Note the cartilage in the sclera (white asterisk). Scale bar represents 17 μm.

Fig 1 shows a cross-section through the eye of the European eel. Going from outside to inside, the sclera, the choroid, the pigment epithelium and the retina can be identified. At first glance, the eye of the European silver eel does not differ from the general organization of the vertebrate eye. However, one striking deviation from this general scheme is present in the posterior eye segment. The eye of the European silver eel has a scleral cartilage (Fig 1, asterisk), covering the whole scleral capsule.

### Mΰller cells

**Fig 2:**
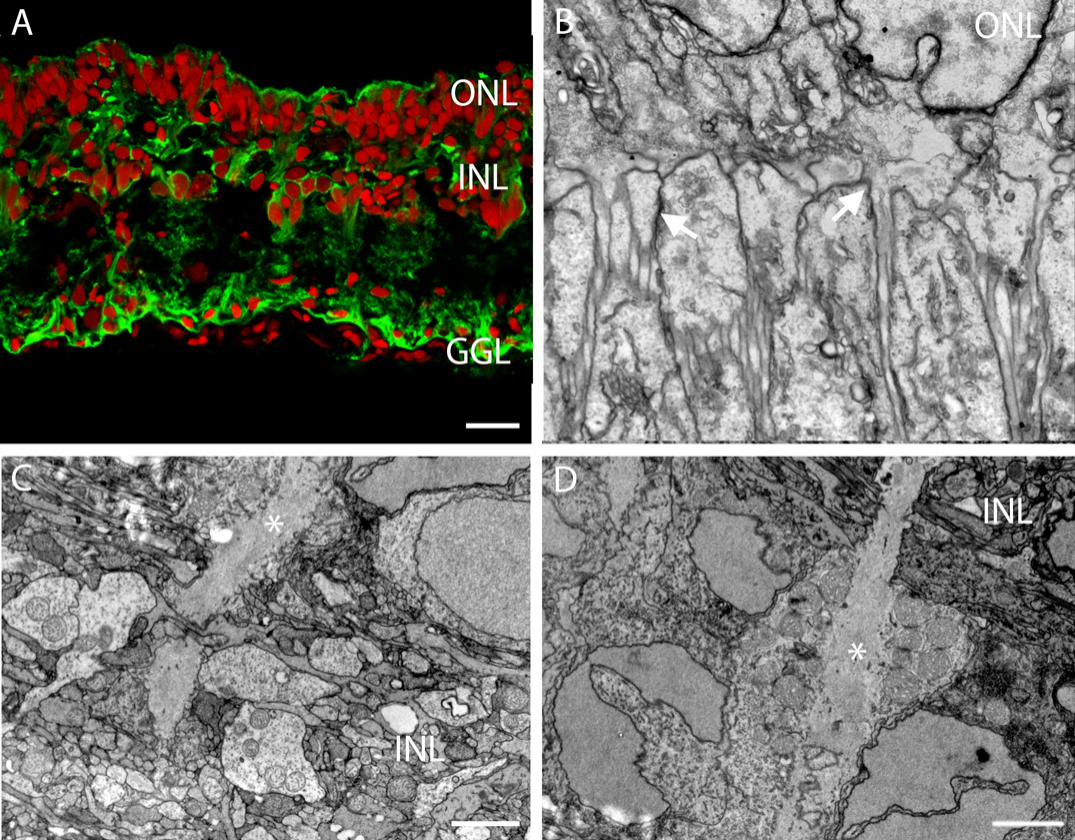
Abundant presence of Mΰller cells in the retina of the European silver eel. A) Confocal picture of the glutamine synthetase immunoreactivity (green). The red label is a nuclear stain. All layers of the retina show thick glutamine synthetase immunoreactive processes. B) Electronmicrograph of the retinal ONL. Junction are made by Mΰller cell processes and photoreceptor processes (white arrows). C) Electronmicrograph showing a Mΰller cell (white asterisk) containing mitochondria at the level of the IPL. D) Electronmicrograph showing a Mΰller cell process (white asterisk) at the level of the INL. Note that this process contains many mitochondria. Scale bar in Fig 2A represents 20 μm, scale bar in Fig 2B represents 1 μm, and scale bars in Fig 2C and 2D represent 2 μm.

Glia cells contain the enzyme glutamine synthetase, which is involved in recycling the neurotransmitter L-glutamate and so protects neurons against excitotoxicity. Mΰller cells are the glia of the retina and perform a crucial task in maintaining stability of the extracellular environment. In the retina of the European silver eel, the glutamine synthetase antibody robustly labelled Mΰller cells (Fig 2A). Protrusions of Mΰller cells in all retinal layers show glutamine synthetase immunoreactivity. In the INL broad glutamine synthetase immunoreactivity is observed and glutamine synthetase positive somata are present in the middle of this layer (Fig 2A). At the ultrastructural level Mΰller cell processes form junctions (arrows) with photoreceptor processes at the level of the outer limiting membrane (Fig 2B). In addition small Mΰller cells processes could be observed (Fig 2B). Broad Mΰller cell processes (asterisks), entering the INL (Fig 2C) or leaving the INL (Fig 2D), seems to contain several mitochondria.

### Photoreceptors

**Fig 3:**
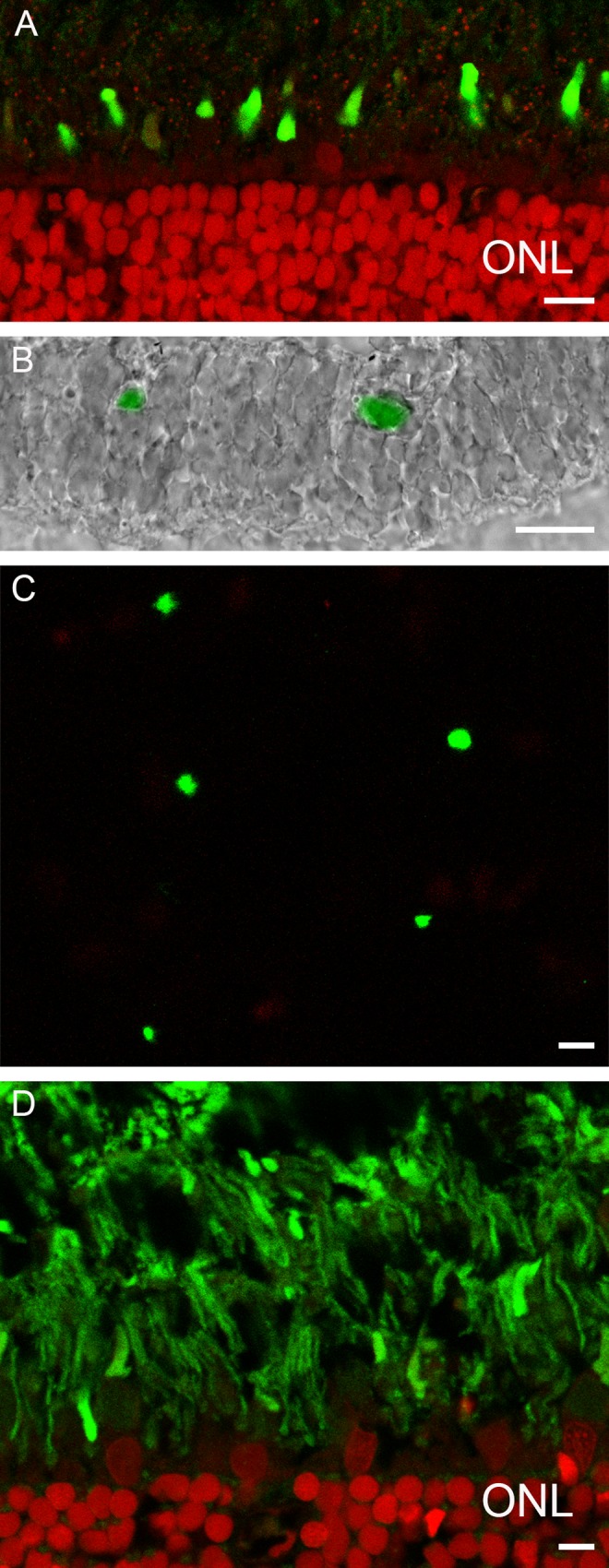
Confocal pictures of the retina of the European eel stained with the M-opsin and the rhodopsin antibodies. A) M-opsin labeling of cone outer segments in a section of the retina (M-opsin: green; Nuclei: red). B) Combined picture of lightmicroscopical image and fluorescence image showing that only cones have M-opsin immunoreactivity. C) M-opsin labeling (green) in a flat mounted section of peripheral retina. D) Rod rhodopsin labeling (green; nuclei: red) is abundantly present in the retina of the European eel. Scale bars in A, C and D represent 5 μm and in B represents 6 μm.

**Fig 4:**
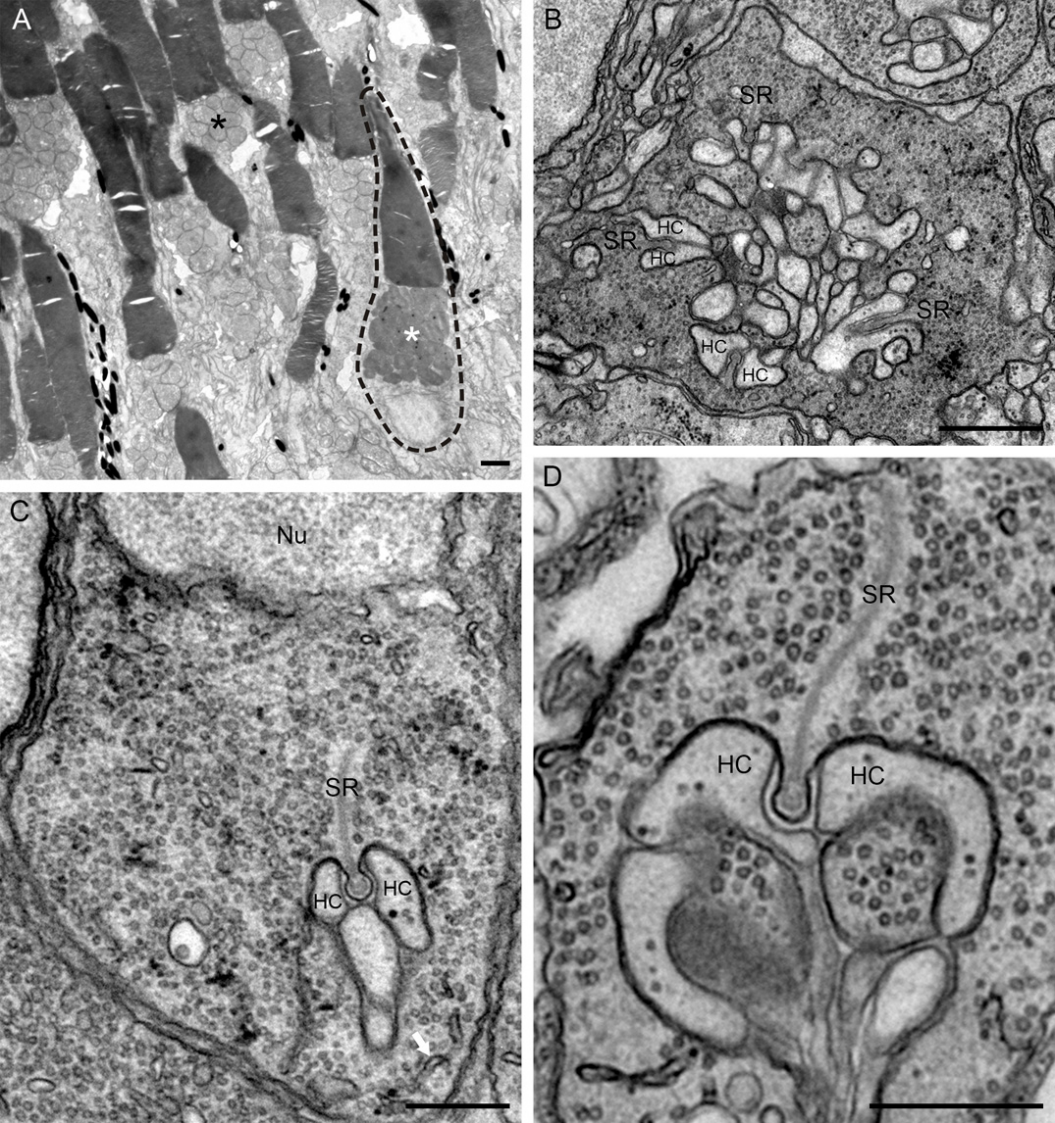
Electronmicrographs of the retina of the European eel. A) The outer and inner segments of the photoreceptors. A cone is encircled with a dotted line while the remained are rods. Note that the mitochondria in the cone inner segments are electron dense (white asterisk) whereas they are electron lucent in rod inner segments (black asterisk). B) Cone pedicle with several ribbon synapses (SR). C) and D) rod spherules (Nu: Nucleus). The white arrow in C (lower right corner) points to the smooth endoplasmic reticulum. The lateral element of the synaptic triad representing horizontal cell dendrites (HC) can transfers to central element. Bars in panels A and B represent 1 μm, bars in panels C and D represent 0.5 μm.

Cone photoreceptors can easily be distinguished in the proximal part of the photoreceptor layer. Cones are arranged in a single layer below the rods. Their outer segments have a cone like structure and their inner segments have a dark appearance (Fig 1, white arrows). To determine which opsins are expressed by the European silver eel we stained the retina with antibodies against the zebrafish L-, M-, S-, UV-opsins and rhodopsin and found that the cone outer segment were only immunoreactive for the M-opsin (Fig 3A, 3B and 3C). No L-, S- or UV-opsin-immunoreactivity was evident. In a combined experiment with lightmicroscopy and fluorescence only cones show M-opsin immunoreactivity (Fig 3B). Using the rhodopsin zebrafish antibody, resulted in abundant immunoreactivity (Fig 3D). In fish retina cones are typically organized in a mosaic, where double cones (L- and M-cones) are accompanied by S- or UV-cones [23,24]. This is not the case for the European silver eel as the M-cones are distributed over the retina in a solitarily fashion and are surrounded by rods (Figs 3B, 3C and 4A).

At the ultrastructural level cones and rods differ markedly in their appearance. Cones have electron dense mitochondria in their inner segments (Fig 4A, white asterisks), while the mitochondria in rods are electron lucent (Fig 4A, black asterisks). In fish retina L-, M-, and S-cone pedicles can be distinguished based on their size [25,26]. L-cone pedicles are large, M-cone pedicles are medium sized and S-cone pedicles are the smallest of the three cone pedicle types. In the OPL of the European silver eel all cone pedicles have a similar size, suggesting that only one cone type is present, corroborating the finding that only M-opsin could be detected.

The general ultrastructural feature of the photoreceptor synaptic terminal is the synaptic triad. Central in the triad is the synaptic ribbon which is surrounded by processes of horizontal and bipolar cells. The lateral elements of the ribbon synapse are horizontal cell dendrites and the central element is a horizontal or bipolar cell dendrite [27]. This classic arrangement is also found in the retina of the European silver eel. Cone pedicles have several triads (Fig 4B) while rod spherule have only one triad (Fig 4C and 4D). In fish retina, the lateral element of a triad can fold such that they become a central element [28], a feature also present in the European silver eel (Fig 4D). The horizontal cell dendrites of the European silver eel contain very few electron dense vesicles. Smooth endoplasmic reticulum can be observed in the rod spherule (Fig 4C, white arrow) but it is not clear whether it is present in the cone pedicle.

Next we focus on the two cell types postsynaptic of the photoreceptors: horizontal and bipolar cells. Although ultrastructure of the European eel retina has been studied in the past [29], no ultrastructural data are available about the synaptic proteins used in the OPL.

### Horizontal cells

**Fig 5:**
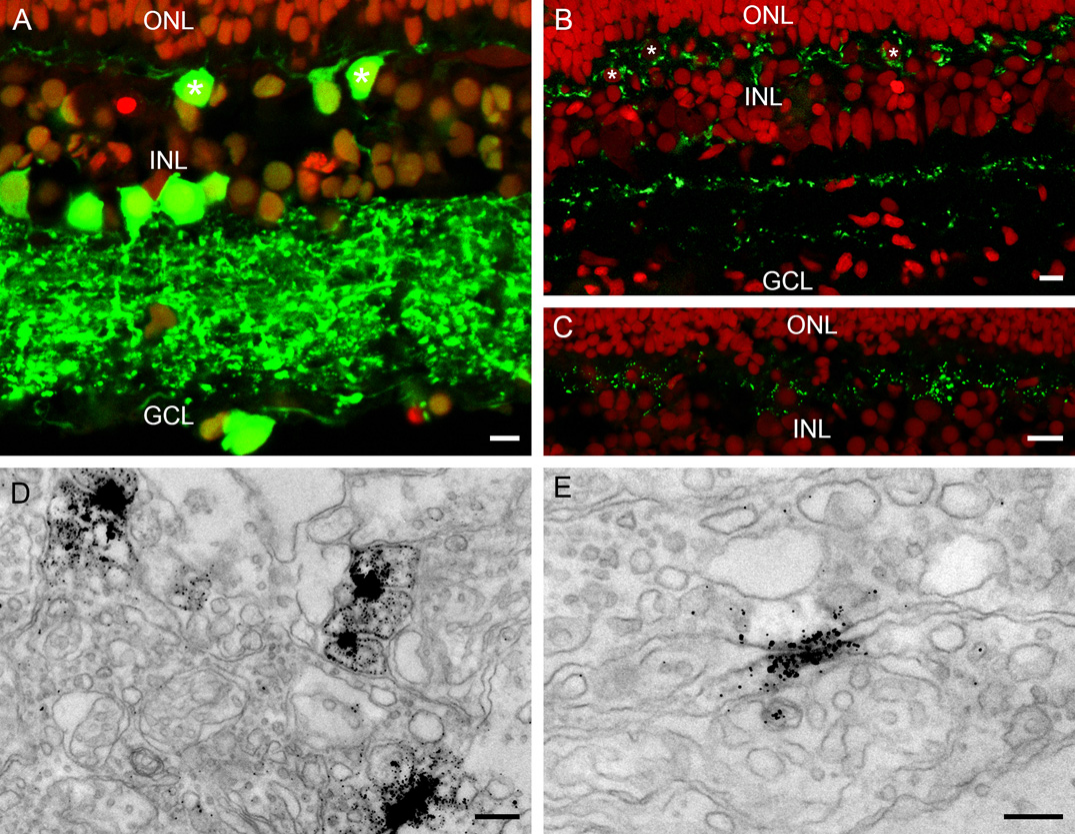
Combined panel of confocal pictures and electronmicrographs focused on horizontal cells. A) Immunofluorescence of the calretinin antibody (green; red: nuclei). Strong labeling occurred in the IPL and some amacrine cells were also labeled. Note, however, the labelling in the horizontal somata and the dendrites (white asterisks). B) Horizontal cells (red: nuclei; white asterisks) are surrounded by TH-immunoreactive processes (green). Additionally, the TH-immunoreactivity in the IPL is indicative of interplexiform cells. C) Punctate labeling of Cx53.8 (green) around the somata of horizontal cells at the level of the OPL. D) and E) Electronmicrographs of the Cx53.8 immunoreactivity. D) Lower magnification of the neuropil of the OPL of the European silver eel retina, showing many Cx53.8 immunoreactive gap junctions. E) Higher magnification of a gap junction. Note that the membranes are separated at the borders of the gap-junction and then come very close together at position of the gap junction itself. At that location the labeling is the strongest. Scale bars in panels A and B represent 5 μm, scale bar in panel C represents 10 μm and scale bars in panel D and E represent 0.25 μm.

In fish retina there is no general marker for horizontal cells, however horizontal cells of the European silver eel are calretinin-immunoreactive (Fig 5A, white asterisks). They form a single layer directly below the photoreceptors. Based on their calretinin immunoreactivity there were no distinct morphological differences observed suggesting that only one type of horizontal cell is present. This is consistent with previous research that found only one functional type of horizontal cell [9] which seem to receive both rod and cone inputs.

In fish, horizontal cells receive a dopaminergic input from interplexiform cells. Dopamine plays an important role in the circadian control of the electrical coupling of horizontal cells. Tyrosine hydroxylase is a precursor enzyme in the cascade of dopamine. Tyrosine hydroxylase immunoreactivity was found at horizontal cell level where it surrounded horizontal cells (Fig 5B). In the IPL two bands of tyrosine hydroxylase immunoreactivity were also found. One tyrosine hydroxylase immunoreactivity band was near the INL while the other weaker band was near the GCL (Fig 5B).

Horizontal cells are coupled by gap junctions [16]. The molecular weight of the gap-junction forming connexins expressed by the horizontal cells in different species varies but is typically around 50 kDa [30]. A general marker for horizontal cell gap junctions is an antibody against the carp Cx53.8 which when used on the European silver eel retina resulted in strong punctate labeling surrounding the horizontal cells (Fig 5C). On the ultrastructural level these puncta can be identified as gap junctions (Fig 5D). The general feature of a gap junction at the ultrastructural level is that the two membranes are in very close contact (Fig 5E). Cx53.8 immunoreactivity is present at both membranes at the point of closest contact.

**Fig 6:**
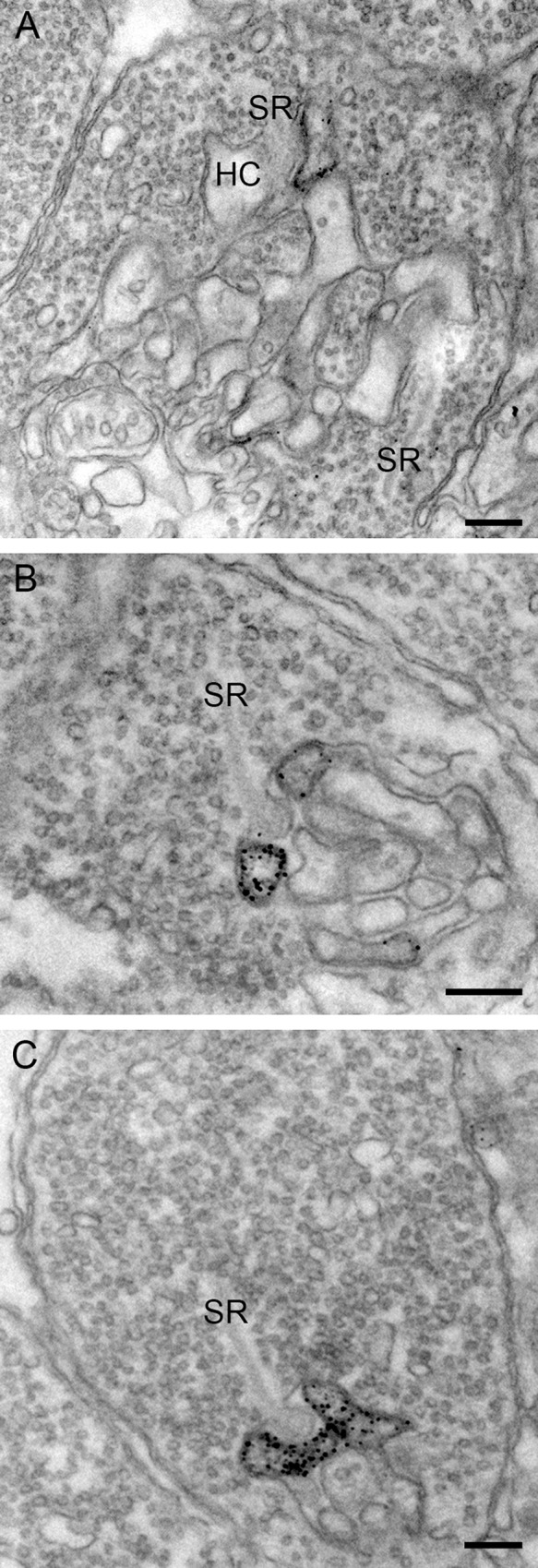
Electronmicrographs of the CX53.8 immunoreactivity at the cone pedicle and the rod spherule level. A) Cone pedicle with Cx53.8 immunoreactivity at one laterally ending horizontal cell dendrite. Note that the opposing horizontal cell dendrite is not labeled. B) and C) Rod spherules with Cx53.8 immunoreactivity in the laterally ending horizontal cell dendrites. In rods, both lateral elements show Cx53.8 immunoreactivity. Synaptic ribbon (SR); horizontal cell (HC). Scale bars represent 0.25 μm.

Connexins can also form hemichannels, which can function as individual entities [31]. Hemichannels present on HC dendrites play a crucial role in the feedback of horizontal cells to cones [18]. In the European silver eel Cx53.8 immunoreactivity is also present in the horizontal cell dendrites ending laterally of the synaptic ribbon in both cones (Fig 6A) and rods (Fig 6B and 6C). Cx53.8 immunoreactivity was present in both lateral elements in the rod spherule whereas for the cone pedicle only one lateral element show Cx53.8 immunoreactivity.

### Bipolar cells

**Fig 7:**
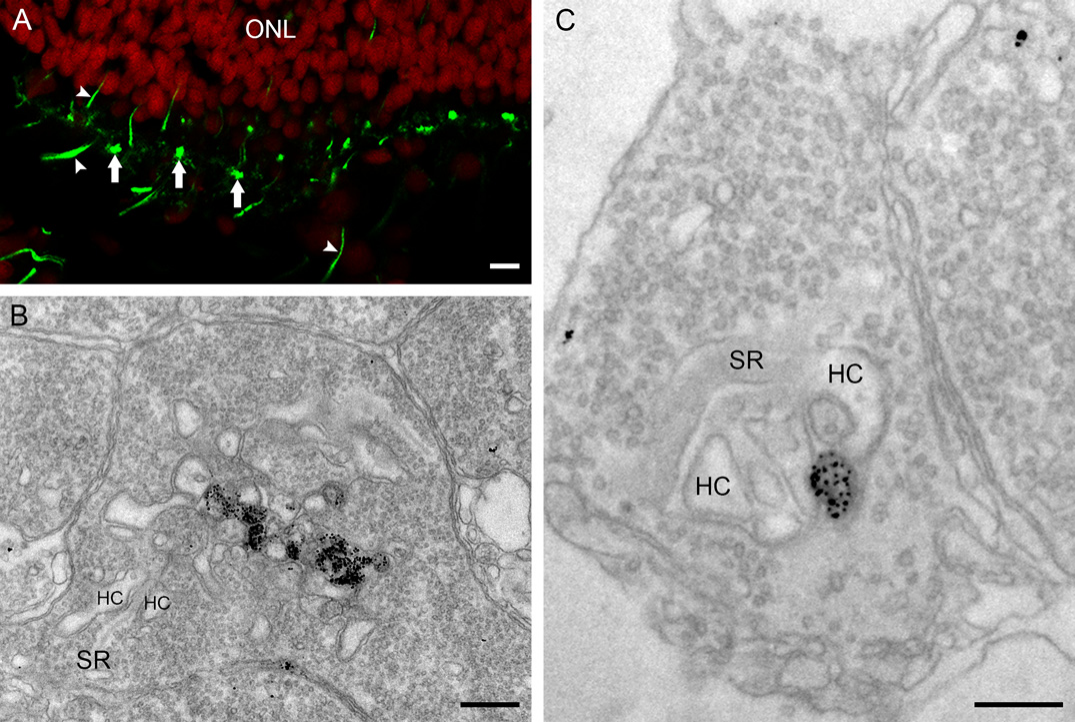
Combined panel of confocal picture and electronmicrographs of the GluR4 immunoreactivity. A) Confocal picture of GluR4 immunoreactivity (green; nuclei: red) in the OPL of the European silver eel retina. Note the punctate labeling of the GluR4 immunoreactivity (white arrows). Mϋller cells also show GluR4 immunoreactivity (white arrowheads). B) Electronmicrograph of a cone pedicle; GluR4 immunoreactivity is seen at the base of the cone pedicle whereas the lateral elements were not labeled. C) Electronmicrograph of a rod spherule. GluR4 immunoreactivity was found in the central element representing the OFF-bipolar cell dendrite. Synaptic ribbon (SR); horizontal cell (HC). Scale bar in panel A represents 5 μm, and scale bars in panel B and C represent 0.25 μm.

In the retina of the European silver eel, ON- and OFF-bipolar cells have been functionally described [9]. The ionotropic glutamate receptor of OFF-bipolar cells in the fish retina consists of GluR4 subunits [32–34]. Consistent with this we found strong punctate GluR4-immunoreactivity in the OPL of the European silver eel retina (Fig 7A, white arrows) suggestive for bipolar dendrites invaginating the cone pedicels. Analysis of the ultrastructure shows that the punctate labeling in the OPL corresponds with OFF-bipolar cell processes invaginating the photoreceptor terminals. GluR4-immunoreactivity was found at the base of the cone pedicle in small profiles whereas the lateral elements representing the horizontal cell dendrites innervating the cone pedicle showed no GluR4-immunoractivity (Fig 7B). For the rod spherule, GluR4-immunoreactivity was only found in the central elements and not in the lateral elements of the triad (Fig 7C). In addition, GluR4-immunoreactivity was found on long structures in the OPL. Such structures are indicative of Mϋller cell processes (Fig 7A, white arrowheads), which can also be observed at the ultrastructural level (not shown).

**Fig 8:**
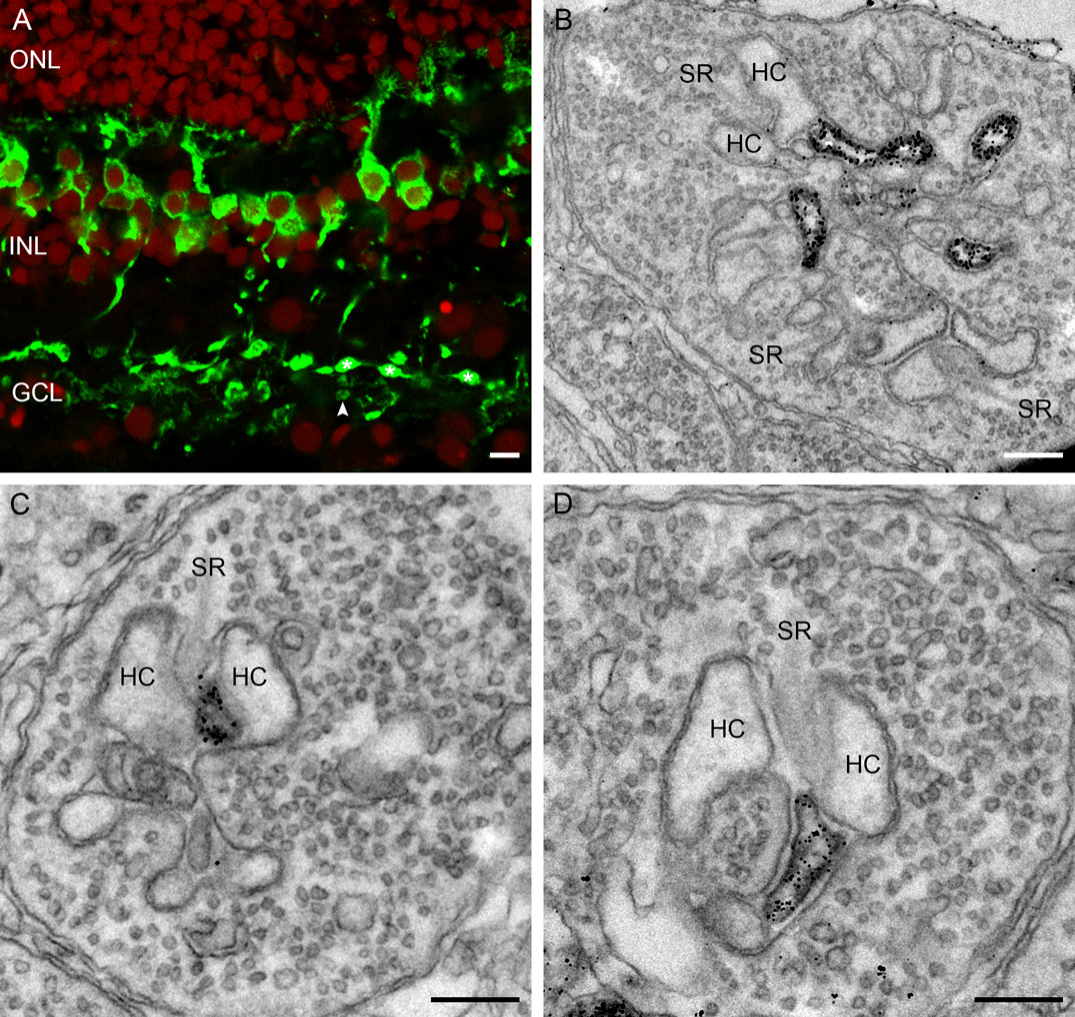
Confocal picture and electronmicrographs of the PKCα immunoreactivity. A) Confocal picture of PKCα immunoreactivity (green; red: nuclei). Note that the entirety of the ON-bipolar cells are labeled from the dendrites in the OPL via the cell somata in INL to the axon terminals in the IPL. Two types of axon terminal show PKCα immunoreactivity, one type is more bulbous (small white asterisks) while the other is smaller (white arrowhead). B) Electronmicrograph of a cone pedicle with PKCα immunoreactivity at the position of the central elements representing ON-bipolar cell dendrites. C) and D) Electronmicrographs of rod spherules with PKCα immunoreactivity at the position of the central elements representing the ON-bipolar cell dendrite. No labeling was found in the lateral elements. Synaptic ribbon (SR); horizontal cell (HC). Scale bar in panel A represents 5 μm, and scale bars in panels B, C and D represent 0.25 μm.

A marker for ON-bipolar cells in the fish retina is PKCα [35] which stains the mixed input and the cone driven ON-bipolar cells. The retina of the European silver eel is no exception. Strong PKCα-immunoreactivity was found in the middle of the INL where bipolar cell somata are located. The thick primary dendrites branch in small dendritic protrusions invaginating the photoreceptor synaptic terminals in the OPL. In the ON-sublamina of the IPL, two types of axon terminals show PKCα-immunoreactivity. One type has a large bulbous axon terminal representing the mixed ON-bipolar cell (Fig 8A, white asterisk), while the cone ON-bipolar cell has a small axon terminal (Fig 8A, white arrowhead). At the ultrastructural level, PKCα-immunoreactivity was found at the central element of the triad, in both cone pedicle (Fig 8B) and rod spherule (Fig 8C and 8D). No PKCα-immunoreactivity was seen at the horizontal cell dendrites in the triad of cones (Fig 8B) or rods (Fig 8C and 8D).

**Fig 9:**
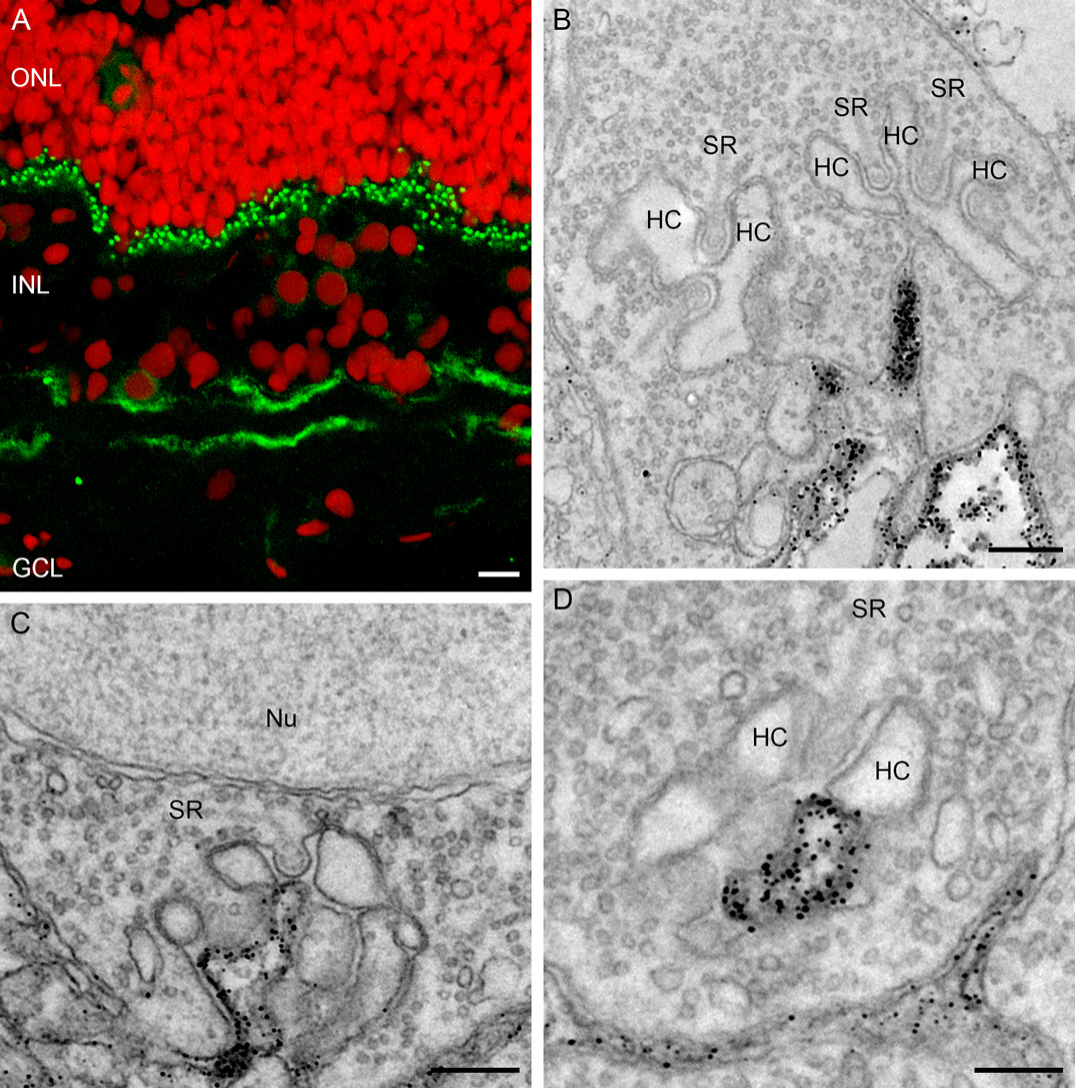
Panel of a confocal picture and electronmicrographs of the mGluR6 immunoreactivity. A) Confocal picture of the mGluR6 immunoreactivity (green; red: nuclei) showing punctate labeling the OPL and two diffuse bands in the IPL. B) Electronmicrograph of a cone pedicle. mGluR6 immunoreactive processes were observed at the base of the cone pedicle. No mGluR6 immunoreactivity was found at the lateral elements representing the horizontal cell dendrites. C) and D) Electronmicrographs of rod spherules showing the mGluR6 immunoreactivity on the central elements of the triad representing the bipolar cell dendrites. Synaptic ribbon (SR); Horizontal cell (HC); Nucleus (Nu). Scale bar in panel A represents 5 μm, bars in panels B, C and D represent 0.25 μm.

**Fig 10:**
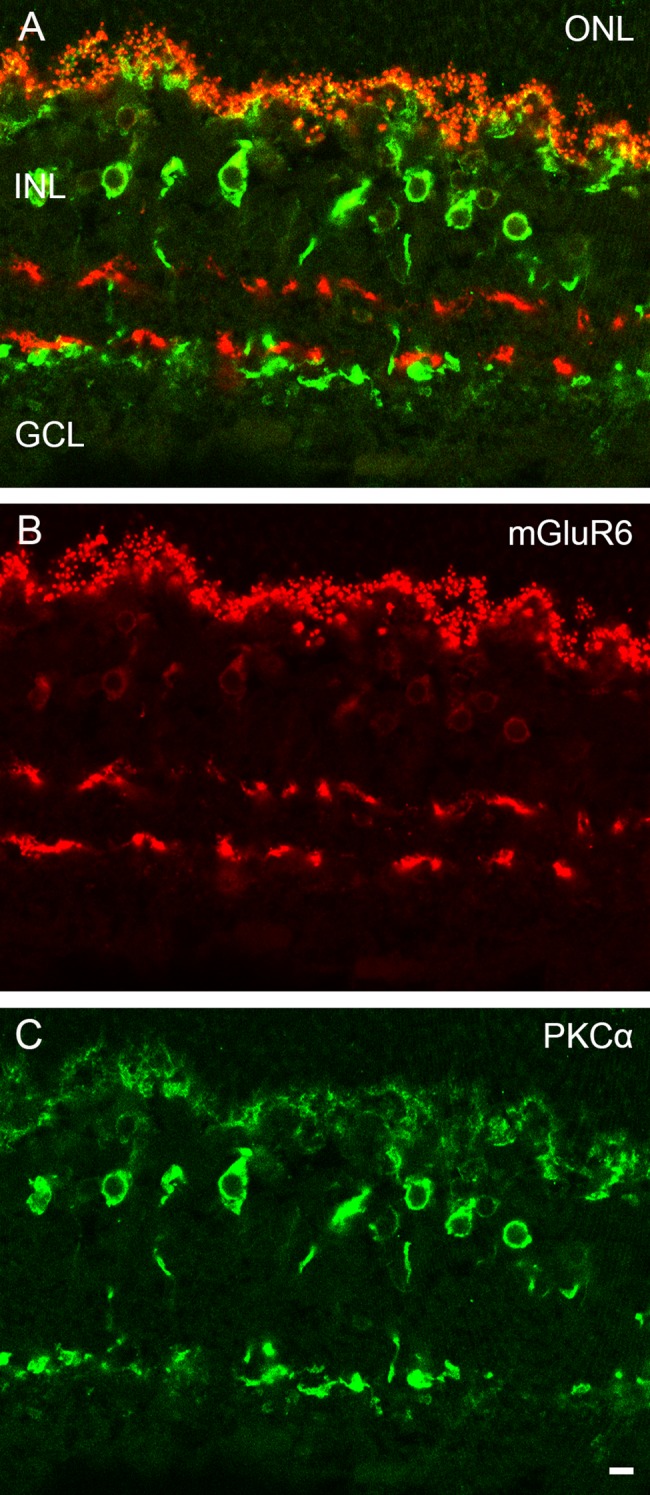
Confocal pictures of double label experiments with the antibodies against mGluR6 and PKCα. A) Retinal section double labeled with antibodies against mGluR6 (red) and PKCα (green) shows co-localization. B) mGluR6 immunoreactivity (red). C) PKCα immunoreactivity (green). Note the clear punctate co-localization in the OPL. No co-localization in the IPL. Scale bar represents 5 μm.

The major metabotropic glutamate receptor in outer retina is mGluR6 [21]. Labeling the retina of the European silver eel with an antibody against the zebrafish mGluR6 resulted in a similar pattern to that found in the zebrafish retina [27]. There was punctate mGluR6-immunoreactivity in the OPL, weak somatic labelling in INL and, two bands of mGluR6-immunoreactivity in the IPL: one band in the OFF- and one in the ON-sublamina (Fig 9A). At the ultrastructural level mGluR6-immunoreactivity was found at both the base of the cone pedicle and at invaginating elements (Fig 9B) and at the central element of the triad in the rod spherule (Fig 9D), indicative for ON-bipolar cell dendrites. No mGluR6-immunoreactivity was found lateral elements of the triads in both cones and rods, indicating that horizontal cells do not express mGluR6. To determine whether all PKCα immunoreactive cells express mGluR6, a double labelling experiment was performed. All PKCα immunoreactive somata in the INL also show mGluR6 immunoreactivity (Fig 10), suggesting that ON-bipolar cells in the retina of the European silver eel express mGluR6. Clear punctate co-localization was found in the OPL (Fig 10A). In the ONL the mGluR6 immunoreactive somata also show PKCα immunoreactivity (Fig 10A). The PKCα immunoreactivity is covering the whole soma, whereas the mGluR6 immunoreactivity only partly covers the somas. In the IPL mGluR6 and PKCα immunoreactivity are not co-localized suggesting that mGluR6 in the IPL also present in some other cell types.

## Discussion

In the present study we studied the outer retina of the European silver eel by immunohistochemical means at both the light and electronmicroscopical level. Only one class of cones was found, which expressed M-opsin, and rods that expressed rhodopsin. OFF-bipolar cell dendrites showed GluR4 immunoreactivity while ON-bipolar cell dendrites showed co-localization of PKCα and mGluR6 immunoreactivity. Both immunopositive OFF- and ON-bipolar cell dendrites innervate the cone pedicle and rod spherule. Horizontal cells were coupled by gap-junctions composed of a connexin homologue to Cx53.8 and these connexins also formed hemichannels at the dendrites of horizontal cells innervating the cone pedicle. Tyrosine hydroxylase immunoreactivity was in close proximity of horizontal cells.

### Opsin expression

The first metamorphosis of the European eel, where it transforms from the larval to glass eel stage, is a true metamorphosis involving drastic morphological changes. In contrast, the morphological changes are much less dramatic for the secondary metamorphosis from the yellow to the silver eel stage [36]. This secondary metamorphosis of the eel is similar to salmon smoltification. During the transformation from yellow eels to silver eels the eye diameter and retinal surface increases [37]. One could speculate that during this fast process, strengthening of the eyeball by means of cartilage could be important.

During its life time the European eel changes its opsin gene expression several times. There are probably two pathways for the changes in opsin gene expression. One way would be to generate new photoreceptors. Mΰller cells are associated with clusters of proliferating retinal progenitor cells that are restricted to the rod photoreceptor lineage [38]. In the present paper it is clear that Mΰller cells are abundantly present in the retina of the European silver eel and it in that way newly formed rods could play a role in the adaptation to the new light environment.

Another way to change the properties of photoreceptors is to switch the opsin, in rods [39], and in cones. It is not fully clear whether there is a real shift in opsin or whether cones are formed anew [40]. In eel, rods switch from fresh water opsin to deep sea opsin [39] and in rainbow trout single S-cones switch from expressing SWS1 to SWS2 opsin [41]. In the European glass eel, cones express RH2 opsin in the ocean, but when they move to rivers they also express SWS2 opsin [40]. For silver eels and larger yellow eels the majority of their cones express RH2 opsin whereas ˂5% of the cones express SWS2 opsin [40].

In the present study we confirm the abundant expression of M-cone opsin in the retina of the European silver eel. However, we could not confirm the sparse expression of the S-opsin in cones. This may be because we used antibodies raised against the zebrafish opsins but we feel that this is unlikely. In general S- and UV-cones are smaller than M-cones and at the ultrastructural level all the cones observed were of approximately equal size. This observation, combined with the opsin expression data, support the notion the European silver eel has only one class of cone photoreceptor, a mid-wavelength sensitive cone.

### Horizontal cells

In all vertebrates, horizontal cells are strongly coupled by gap junctions. The European silver eel is no exception. We found a similar pattern of connexin expression as seen other fish. Abundant punctate labeling between horizontal cells is indicative of the strong electrical coupling by gap-junctions and small punctate labeling at the tips of the horizontal cell dendrites indicates the presence of connexin-hemichannels, which are involved in negative feedback from horizontal cells to cones [19,20]. In the triads in the cones synaptic terminal, only one lateral element expresses Cx53.8 hemichannels. As these lateral elements represent the dendrites of horizontal cells, it may suggest that there is more than one type of horizontal cell present in the European eel retina. Alternatively, it could be that the amount of Cx53.8 hemichannels is strongly regulated to fine tune the strength of negative feedback from horizontal cells to cones. Coupling of horizontal cells is modulated during light-adaptation [42] via dopamine released by interplexiform cells onto horizontal cells. The strong expression of tyrosine hydroxylase in the close proximity of the soma of the horizontal cells shows that a similar mechanism is present in the European silver eel.

### OFF-pathway

The glutamate receptor subunit of the OFF-bipolar cells in the European silver eel is GluR4, just as in goldfish, zebrafish and carp [33–35,43]. The glutamate receptors are localized on the tips of the OFF-bipolar cell dendrites invaginating the photoreceptor synaptic terminals and ending close to the synaptic ribbons. The presence of GluR4 immunoreactivity for OFF-bipolar cells is supported by data showing that photoreceptor input to OFF-bipolar cells is mediated by AMPA/kainate receptors [44–46]. In contrast to mammals GluR4 is absent from the dendrites of horizontal cells [47,48]. In fish horizontal cells express GluR2 [32,49]. We were unable to confirm this for the European eel, presumably our GluR2 antibody was not raised against the native protein.

### ON-pathway

In vertebrates ON-bipolar cells all express PKC. PKCα labeling in fish shows two types of bipolar cells: one bipolar cell type receiving exclusively cone input and one bipolar cell type receiving rod and cone input [35]. A similar arrangement is found in the European silver eel. The rod input to mixed input bipolar cells in fish retina is sensitive to L-(+)-2-amino-4-phosphonobutryric acid (APB) and mediated by a Group III metabotropic glutamate receptors mGluR6 [44,50,51]. In fish the cone input to the ON-bipolar cells is mediated by a glutamate transporter [52]. In the present study we demonstrated the presence of mGluR6 in the retina of the European silver eel, on the dendrites of ON-bipolar cells contacting both rod and cones. The cone contacts are at the base of the cone pedicle whereas the rod contacts are invaginating contacts ending close to the synaptic ribbon.

The two bands in the IPL of mGluR6 immunoreactivity is in agreement with results found for zebrafish retina [21]. Both species show mGluR6 immunoreactivity in both the ON- and OFF-layer of the IPL. There are precedents for mGluR6 expression in the inner retina. Using *in situ* hybridization techniques in the retina of zebrafish larvae [21] and in the human retina [53] shows the presence of mGluR6 encoding mRNA in ganglion cells.

### In Conclusion

The synaptic organization of the OPL of the European silver eel shows a great similarity with the OPL of cyprinid fish. All the key components for the ON- and OFF-pathways, center/surround organization and feedback pathway from horizontal cells to cones are present. The most striking differences is the presence of only one cone-type.
